# Exploring the role of directly coupled alternating electric fields on chondrocyte morphology and redifferentiation capacity with a focus on sex differences

**DOI:** 10.1002/jeo2.70261

**Published:** 2025-05-06

**Authors:** Zezhong Song, Vivica Freiin Grote, Franziska Sahm, Julius Zimmermann, Christoph Lutter, Anika Jonitz‐Heincke, Rainer Bader

**Affiliations:** ^1^ Department of Orthopaedics, Biomechanics and Implant Technology Research Laboratory Rostock University Medical Center Rostock Germany; ^2^ Institute of General Electrical Engineering University of Rostock Rostock Germany; ^3^ Department Life, Light Matter University of Rostock Rostock Germany

**Keywords:** articular cartilage lesion, cell‐based therapy, chondrocyte redifferentiation, directly coupled electrical stimulation

## Abstract

**Purpose:**

In cell‐based therapies addressing articular cartilage lesions, a central challenge is to avoid the formation of fibrous cartilage resulting from dedifferentiation processes. Electrical stimulation emerges as a promising approach for promoting chondrocytic redifferentiation. This study investigated the effects of varying electric fields on morphological changes and the redifferentiation capacity of human chondrocytes with regard to alterations in sex.

**Methods:**

Chondrocytes, isolated from the articular cartilage of male and female patients undergoing total knee replacement, were exposed to alternating electric fields of varying strengths ranging from 0.8 to 1.2, 15 to 20 and 100 to 140 V/m. Afterwards, cell morphology and viability, as well as the deposition of collagen (Col) 1 and 2, were evaluated.

**Results:**

Following electrical stimulation, in particular at 15–20 V/m, an increase in the Col2/Col1 ratio and an elevated proportion of rounded, chondrocyte‐like cell morphology were observed, indicating a promoting effect on the redifferentiation of chondrocytes. Comparative analysis between both sexes revealed that chondrocytes from female donors exhibit higher Col1 synthesis rates, a decreased Col2/Col1 ratio, and a higher proportion of elongated, fibroblast‐like cells compared to chondrocytes derived from male donors.

**Conclusion:**

Our in vitro study suggests that chondrocytes from male donors are more prone to re‐differentiate after electrical stimulation.

**Level of Evidence:**

N/A.

**Abbreviations**
AC: alternating current
ACI: autologous chondrocyte implantation
CICP: Type I C‐terminal collagen propeptide
CO_2_
: carbon dioxide
Col: collagen
CPII: Type II C‐terminal propeptide
DC: direct current
DMEM: Dulbecco's Modified Eagle Medium
ECM: extracellular matrix
Ex/Em: excitation/emission
FITC: fluorescein isothiocyanate
IKDC: International Knee Documentation Committee
KOOS: knee injury and osteoarthritis outcome
min: minutes
mL: millilitres
mm: millimetre
mRNA: messenger ribonucleic acid
ms: milliseconds
ng/µg: nanograms per micrograms
nm: nanometre
O_2_
: oxygen
OA: osteoarthritis
OD: optical density
PEEK: polyetheretherketone
SD: standard deviation
SOX9: SRY‐box transcription factor 9
V_rms_
: root‐mean‐square voltage
V/m: voltage per metre
WST‐1: water‐soluble tetrazolium salt
(BM‐)MSC: (bone marrow‐derived) mesenchymal stem cells
°C: degree celsius
(k)Hz: (kilo‐)hertz

## INTRODUCTION

Hyaline cartilage is characterized by the absence of blood and lymphatic vessels, the lack of innervation and a low chondrocyte‐to‐matrix volume ratio. These properties result in a limited capacity for self‐repair and regeneration [27]. Articular cartilage is a specialized subtype of hyaline cartilage that is essential for promoting normal joint function. It provides a smooth, lubricated, low‐friction surface and absorbs external pressure during joint movement [16]. Trauma‐induced cartilage lesions and age‐related cartilage degeneration are two prevalent factors that lead to impaired joint function, pain, and ultimately osteoarthritis [8]. Osteoarthritis (OA), the most common form of arthritis, affects more than 500 million people worldwide [32]. Here, sex‐specific differences appear to play a significant role in the natural history of the disease, as it is widely acknowledged that females have a greater predisposition to getting osteoarthritis [40].

To date, the treatment of articular cartilage lesions and degeneration remains a challenge. Notably, autologous chondrocyte implantation (ACI) has emerged as a promising approach for the treatment of cartilage lesions in younger patients [13]. The ACI procedure involves three steps, that is, the isolation of chondrocytes from a non‐weight‐bearing area, in vitro chondrocyte expansion, and the surgical implantation of cultured chondrocytes into the cartilage defect. The implanted chondrocytes are expected to secrete the extracellular matrix (ECM) necessary for fixation within the defect [13]. However, the in vitro expansion process is consistently associated with chondrocyte dedifferentiation [13], which is characterized by the loss of the chondrogenic phenotype [31, 35]. Morphologically, dedifferentiated chondrocytes undergo a transformation from a rounded, chondrocyte‐like cell shape to a fibroblastic morphology characterized by elongated cells [31, 35]. This phenotypic shift results in diminished collagen (Col) 2 and proteoglycan synthesis as well as elevated Col1 expression rates [31, 34, 44]. Implantation of dedifferentiated chondrocytes leads to the formation of fibrocartilage with inferior mechanical properties, failing cartilage repair [9].

To attain the redifferentiation of chondrocytes cultured in vitro, a broad spectrum of research has been initiated, elucidating various relevant factors such as three‐dimensional culture with scaffolds, utilization of growth factors, mechanical stimulation and electrical stimulation [20, 25, 51]. Among these, electrical stimulation has gained increasing attention [51]. It has been demonstrated that articular cartilage exhibits electrochemical properties [21]. The electrochemical characteristics of hyaline cartilage are based on the displacement of unbound cations along the relatively fixed negative charges of the proteoglycans. These properties give rise to phenomena like streaming and diffusion potentials, along with charge‐dependent osmotic swelling pressures [29, 52, 53]. These properties, in turn, elicit external electric signals that translate into intracellular signals, influencing cell proliferation and ECM component formation [52]. Recent research investigating the effects of electrical stimulation on cartilage tissue and chondrocytes has demonstrated increased chondrocyte proliferation, enhanced matrix formation and diminished matrix breakdown [7, 51]. Therefore, electrical stimulation may be beneficial for chondrocyte redifferentiation during the expansion period of ACI.

The objective of this study was to electrically stimulate patient‐derived chondrocytes with a range of varying electric fields by direct coupling to reveal the potential influence of different electric field strengths on chondrocyte morphology and redifferentiation capacity. In this context, comparisons between chondrocytes derived from male and female patients are conducted to explore whether there is a different response to electrical stimulation between the sexes.

## MATERIALS AND METHODS

### Experimental setup for electrical stimulation

**Figure 1:**
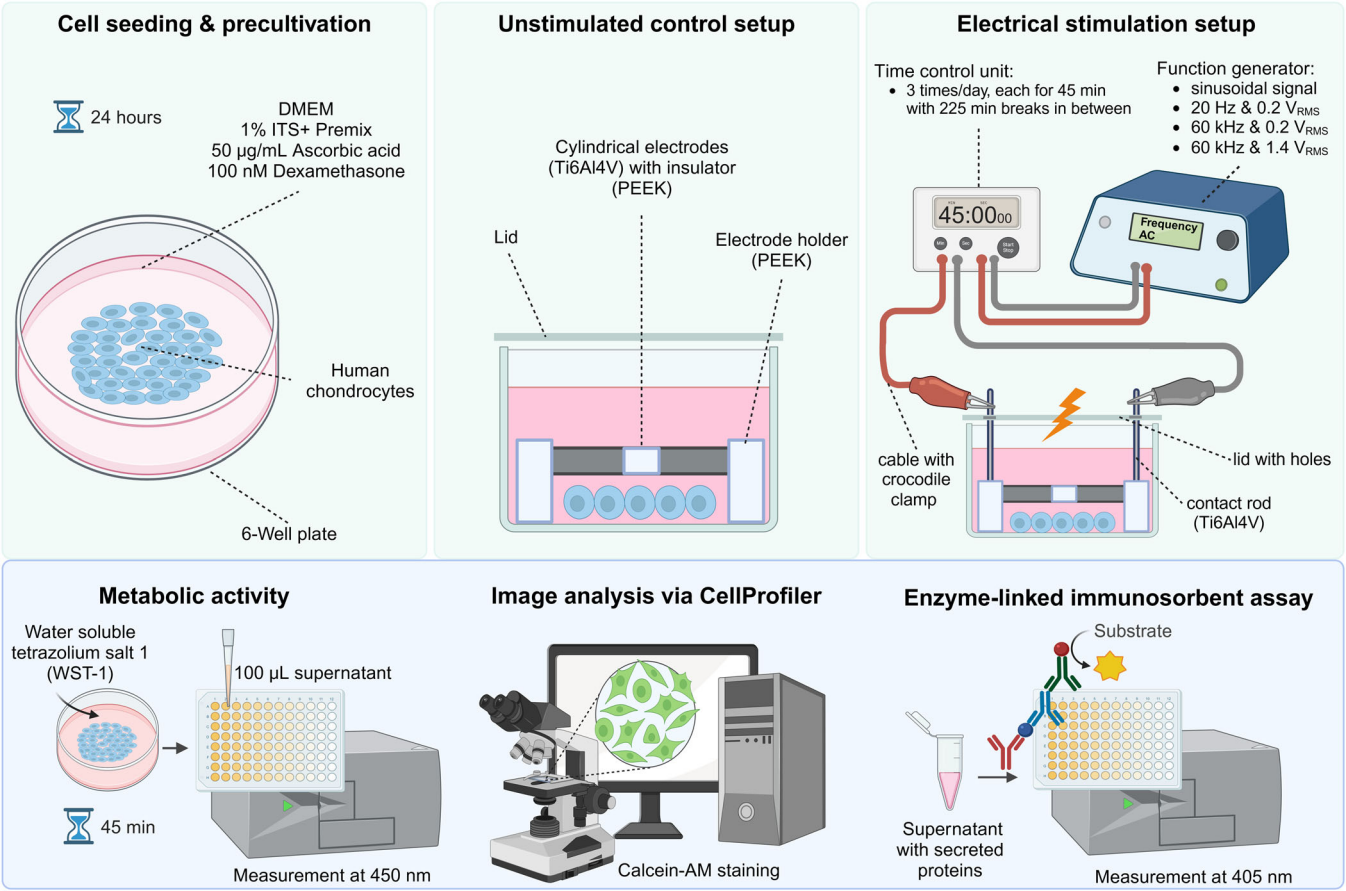
Schematic overview of the experimental design and the electric stimulation setup consisting of two cylindrical electrodes made of a titanium alloy with an insulator in between. The electrical fields within the cell culture were coupled in via a function generator, on which the input voltages and frequency were set. Created in BioRender. Jonitz‐Heincke (2025). https://BioRender.com/z6jx4ix.

The experiments were based on the established setup previously used to investigate the impact of electric fields on human chondrocytes, mesenchymal stem cells and osteoblasts [19, 42]. The cylindrical electrodes, constructed from commercially pure titanium, exhibited dimensions of 14 mm in length and 5 mm in diameter. A 5 mm‐long insulator made of polyetheretherketone (PEEK) separated the electrodes. The electrode holders, also crafted from PEEK, ensured a 3 mm gap between the electrodes and the cell culture well bottom. The contact rods, measuring 35 mm in length, were composed of titanium. The lid of the 6‑well plate had pre‑drilled holes for the contact rods. Chondrocytes were seeded and cultured at the central region of the cell culture well bottom beneath the electrodes with 5 mL medium added. For electrical stimulation, alternating electric fields at 20 Hz and 0.2 V_rms_, 60 kHz and 0.2 V_rms_, as well as 60 kHz and 1.4 V_rms_, were used. Under these combinations, electric potentials inside the stimulation chamber were measured as V_rms_ at defined coordinates at the bottom of the cell culture plate according to the previous study of Zimmermann et al. [54]. By calculating their gradients, the resulting electric field strengths were determined to be 0.8–1.2 V/m (20 Hz, 0.2 V_rms_), 15–20 V/m (60 kHz, 0.2 V_rms_) and 100–140 V/m (60 kHz, 1.4 V_rms_), respectively. A sketch of the entire experimental setup and the electrode design is shown in Figure 1.

### Cell cultivation and stimulation

For the stimulation experiments, human chondrocytes were sourced from six patients (male [*n* = 3]: 64.3 ± 2.1 years; female [*n* = 3]: 72.7 ± 2.6 years) undergoing primary total knee replacement. The study received approval from the Local Ethical Committee of the University of Rostock (registration no. A2009‐17). Human chondrocytes were isolated from the articular cartilage under sterile conditions, following established protocols [24]. Chondrocytes were expanded at 37°C, 5% CO_2_, and 21% O_2_ and cryopreserved at passage 2. Upon thawing, chondrocytes were cultivated in Dulbecco's Modified Eagle Medium (DMEM, Gibco, Thermo Fisher Scientific Inc) with 10% foetal bovine serum (Pan Biotech), 1% penicillin/streptomycin (Thermo Scientific), 1% amphotericin B (Biochrom GmbH) and 50 μg/mL ascorbic acid (Sigma‐Aldrich, Merck KGaA) in a 75 cm^2^ cell culture flask at 37°C, 5% CO_2_, and 21% O_2_ for 7 days to reach a confluence of nearly 90%. Chondrocytes in passage 4 were used for electrical stimulation.

Twenty thousand chondrocytes in the fourth passage were seeded in a 6‐well cell culture plate positioned in the centre of the well. Following an initial adherence period of 45 min, each well was filled with 5 mL DMEM containing 1% penicillin/streptomycin, 1% Amphotericin B, 1% insulin‐transferrin‐selenium (ITS+™ Premix, BD Biosciences), 50 μg/mL ascorbic acid, and 100 nM dexamethasone (Sigma‐Aldrich, Merck KGaA). After 24 h of incubation, the electrical stimulation started. The complete electrical stimulation unit, consisting of the electrodes, holder and contact rods, was placed in the well. The specific alternating electric fields as sinusoidal signals were applied to the stimulation chamber for 3 days using a Metrix GX 310 function generator (Chauvin Arnoux Ltd). This resulted in the electric field strength, as mentioned before. Chondrocytes underwent stimulation three times per day for 45 min each, with 225‐min breaks between stimulations [19]. During stimulation, chondrocytes were cultured at 37°C under 5% CO_2_ and 21% O_2_. For the unstimulated control, chondrocytes were similarly cultured in the presence of the electrode system but without a connection to the function generator (Figure 1).

### Cell staining, microscopy and image analysis

After removal of the medium, chondrocytes were stained with Calcein AM (Thermo Fisher Scientific, Inc.) at room temperature for 10 min. Calcein AM (Ex/Em 494/515 nm) is a cell‐permeable dye that is used for the determination of living cells. In living cells, the nonfluorescent calcein AM is hydrolysed into green fluorescent calcein by intracellular esterases. The stained samples were captured at ×10 magnification using the Nikon Eclipse 120 fluorescence microscope (Nikon Instruments). A FITC filter was used for imaging. A total of four images were acquired for each well. Image acquisition settings for the microscope were configured with a 250 ms exposure time.

Prior to analysis, preprocessing was done using ImageJ 1.53 (National Institutes of Health, available at https://imagej.net/ij/). The brightness/contrast of the images was adjusted, with a minimum displayed value of 0 and a maximum displayed value of 60. Subsequently, chondrocytes that were in close contact with each other were manually separated. The analysis was then performed using CellProfiler (version 4.2.1; Broad Institute) [10, 23, 47]. Chondrocytes were classified according to their shape as either rounded or elongated, with the form factor used as criteria. The form factor is a dimensionless quantity used to describe the shape of a cell: Form factor = 4π × cell area/cell perimeter^2^, where π is the mathematical constant ~3.14 [45]. The form factor has a value of 1 when the cellular outline is a perfect circle and decreases as the cell elongates [38].

### ECM protein synthesis

For quantifying the amount of Type I C‐terminal collagen propeptide (CICP), the MicroVue CICP ELISA (Quidel) was used following the manufacturer's instructions. A standard curve was incorporated to determine protein concentrations within the samples, with absorption measured at 405 nm using a Tecan microplate reader (Tecan Group Ltd.). Similarly, the Col2 Synthesis ELISA (IBEX) was utilized to measure the concentration of Type II C‐terminal propeptide (CPII). The assay procedure was carried out in accordance with the manufacturer's instructions, with absorption measured at 450 nm using a Tecan microplate reader (Tecan Group Ltd.). All protein data underwent normalization to total protein content, which was determined using the Qubit® Protein Assay (Thermo Fischer Scientific Inc.).

### Metabolic activity

The water‐soluble tetrazolium salt (WST‐1) assay (Roche GmbH) was performed to assess the metabolic activity of chondrocytes. Mitochondrial dehydrogenase promotes the reduction of the tetrazolium salt to formazan, changing the colour from orange to yellow. Following the removal of the medium, 500 μL of a 10% dilution of WST‐1 reagent with DMEM was added to each well. Subsequent to an incubation period of 1 hour at 37°C, 100 μL from each well were transferred into a 96‐well plate, maintaining duplicates. The absorption at a wavelength of 450 nm (reference wavelength of 630 nm) was measured and deducted to a blank using the multimode plate reader Infinite 200 pro (Tecan Group Ltd.).

### Data illustration and statistical analysis

The results of the cell experiments were presented graphically using scatter plots created with GraphPad Prism 9.5.1 (GraphPad Software Inc.). The scatterplots depict the individual values, accompanied by essential statistical parameters, including mean values and standard deviations. Statistical analyses were conducted using GraphPad Prism 9.5.1. Data were assessed for normality using the Shapiro–Wilk test. All comparisons between groups were made separately using a two‐sample test. For comparisons without sex‐based distinction and within the same sex, paired *t*‐tests (for normally distributed data) and Wilcoxon matched‐pairs signed rank tests (for non‐normally distributed data) were used. Unpaired *t*‐tests (for normally distributed data) and Mann–Whitney tests (for non‐normally distributed data) were used to compare both sexes. For the statistical analysis of the cell morphology, one‐way analysis of variance (ANOVA) with Tukey's multiple comparison test was used. A *p*‐value < 0.05 indicated statistical significance, while a *p*‐value between 0.05 and 0.1 suggested a statistical trend.

## RESULTS

### Analysis of cell morphology and viability

In total, 19,824 chondrocytes (male, *n* = 9958; female, *n* = 9866) were analysed. The analysis of the form factor revealed both stimulation‐ and sex‐dependent differences. Excluding both sexes, the mean form factor of unstimulated cells was 0.68 ± 0.16. Stimulation with electric fields of 0.8–1.2 and 15–20 V/m increased the form factor for both to 0.70 ± 0.16, respectively, whereas stimulation with 100–140 V/m did not influence the form factor (0.68 ± 0.15) compared to unstimulated cells.

**Figure 2:**
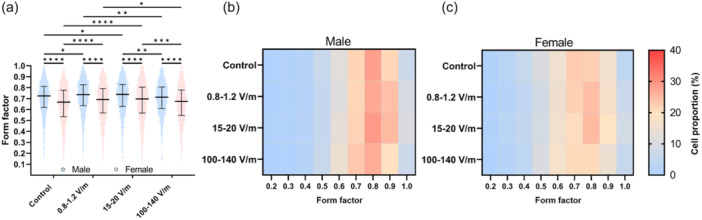
Human chondrocytes were exposed to alternating electric fields of 0.8–1.2, 15–20 and 100–140 V/m for 3 days. Afterwards, cellular morphology was analysed. For this purpose, cells were stained with Calcein AM, and images were captured. The images were analysed using CellProfiler, and the cells were classified into rounded and elongated cells, with form factor as the criterion. (a) Data were presented as individual values with mean and standard deviations. One‐way confidence interval (ANOVA) was conducted for statistical analysis with multiple comparisons using Tukey's multiple comparisons test, *p ≤ 0.05, **p ≤ 0.01, ***p ≤ 0.001, ****p ≤ 0.0001. (b, c) Heatmaps indicate the proportion of cartilage cells (in %) in the corresponding form factor area. The form factor is displayed as the corresponding upper limit of a specific range, for example, 0.3 corresponds to a range of 0.2–0.299(…), and so on. As an exception, 0.2 corresponds to a range of 0.0–0.199(…), and 1.0 corresponds to a range of 0.9 and 1.0.

The form factor of cells from male and female donors is presented in Figure 2a. For all stimulation groups, significant differences were present between both sexes, with cells from males having a higher form factor than those from females (mean ± SD; control: 0.71 ± 0.15 [male]; 0.65 ± 0.17 [female], *p* < 0.0001; 0.8–1.2 V/m: 0.72 ± 0.15 [male]; 0.67 ± 0.16 [female], *p* < 0.0001; 15–20 V/m: 0.72 ± 0.15 [male]; 0.68 ± 0.17 [female], *p* < 0.0001; 100–140 V/m: 0.70 ± 0.14 [male]; 0.66 ± 0.16 [female], *p* < 0.0001). Further, exposure to the different electric fields revealed significant differences for both sexes. For cells from males, chondrocytes significantly exhibited a more rounded phenotype when exposed to 0.8–1.2 V/m (*p* = 0.0437) and 15–20 V/m (*p* = 0.0266) compared to unstimulated cells. Stimulation with 100–140 V/m led to more elongated cells compared to 0.8–1.2 V/m (*p* = 0.0032) and 15–20 V/m (*p* = 0.0016). The same was true for the cells from female donors, with significant differences between control and 0.8–1.2 V/m (*p* < 0.0001) and 15–20 V/m (*p* < 0.0001) as well as between 100–140 and 0.8–1.2 V/m (*p* = 0.0132) and 15–20 V/m (*p* = 0.0005).

In order to further visualize the percentage of cells in the corresponding form factor areas, corresponding heat maps were plotted (Figure 2b,c). In these, blue colouring corresponds to a low percentage of cells, whereas red represents a high percentage of cells. The form factor shown corresponds to a defined range, that is, a form factor of 0.5 represents numbers between 0.4 and 0.499(…), and so on. As an exception, the form factor of 0.2 corresponds to a range of 0–0.199(…), and 1.0 corresponds to a range between 0.9 and 1.0. Only a tiny proportion of chondrocytes from male donors (Figure 2b) exhibited a form factor ranging from 0.2 to 0.4 (control: 9.5%; 0.8–1.2 V/m: 8.0%; 15–20 V/m: 8.9%; 100–140 V/m: 8.8%). An increase in the number of cells is noticeable from 0.6. (control: 12.0%; 0.8–1.2 V/m: 11.5%; 15–20 V/m: 12.8%; 100–140 V/m: 14.3%). Similar to the results of a form factor of 0.6, stimulation with 100–140 V/m led to a higher proportion of cells with a form factor of 0.7 compared to the other stimulations (control: 21.9%; 0.8–1.2 V/m: 21.5%; 15–20 V/m: 21.8%; 100–140 V/m: 23.3%). The highest proportion of cells could be defined with a form factor of 0.8. Stimulation with 15–20 V/m resulted in the highest proportion (control: 28.3%; 0.8–1.2 V/m: 27.1%; 15–20 V/m: 28.9%; 100–140 V/m: 26.6%). Furthermore, an elevated proportion of cells with a form factor of 0.9 could be determined after exposure to 0.8–1.2 and 15–20 V/m (control: 21.4%; 0.8–1.2 V/m: 24.2%; 15–20 V/m: 24.9%; 100–140 V/m: 20.3%).

In contrast to cells from male donors, chondrocytes from female donors (Figure 2c) have a higher proportion of cells in a form factor range of 0.2–0.4 (control: 19.7%; 0.8–1.2 V/m: 15.1%; 15–20 V/m: 16.5%; 100–140 V/m: 16.4%). The percentage of cells with a form factor of 0.6 is also elevated in female donors (control: 15.5%; 0.8–1.2 V/m: 15.1%; 15–20 V/m: 16.0%; 100–140 V/m: 17.0%). Unstimulated cells and cells exposed to the lowest electric field showed a higher proportion at a form factor of 0.7 (control: 22.7%; 0.8–1.2 V/m: 22.1%; 15–20 V/m: 20.1%; 100–140 V/m: 21.0%). Stimulation with 0.8–1.2 V/m and 15–20 V/m caused the highest proportion of cells with a form factor of 0.8 (control: 22.3%; 0.8–1.2 V/m: 24.3%; 15–20 V/m: 25.0%; 100–140 V/m: 21.0%). Compared to chondrocytes from male donors, the proportion of cells from female donors with a form factor of 0.9 was significantly lower. However, exposure to 15–20 V/m led to the highest proportion of cells in this range (control: 15.8%; 0.8–1.2 V/m: 16.9%; 15–20 V/m: 18.9%; 100–140 V/m: 14.4%).

**Figure 3:**
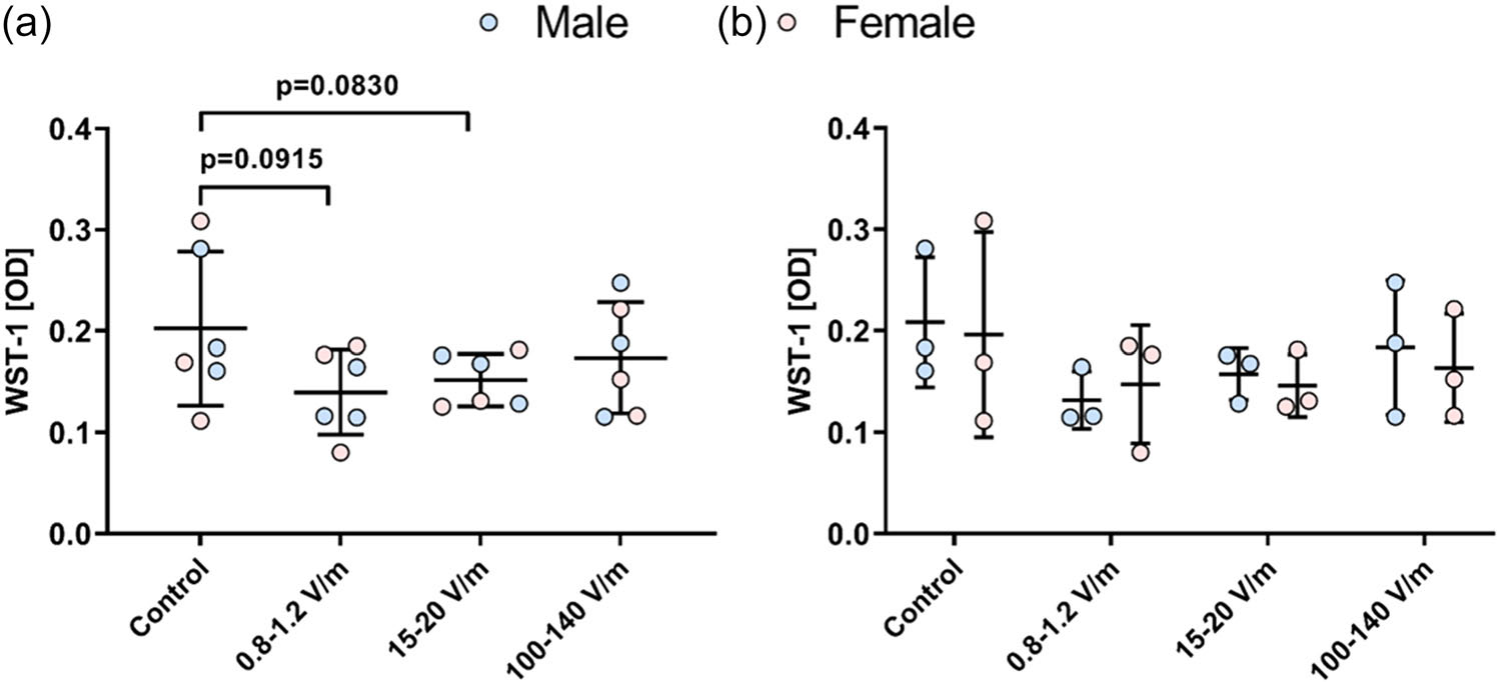
Human chondrocytes were exposed to alternating electric fields of 0.8–1.2, 15–20 and 100–140 V/m for 3 days. Afterward, cellular viability via WST‐1 assay was analysed. (a) Comparison of cell metabolic activity between different groups without sex‐based distinction. (b) Comparison of cell metabolic activity both within and between chondrocytes from male and female donors. The optical density (OD) data were presented as individual values (male [*n* = 3]; female [*n* = 3]) with means and standard deviations. Statistical analysis was conducted by (a) paired *t*‐test, and (b) Wilcoxon matched‐pairs signed rank test (comparison within the same sex) and Mann–Whitney test (comparison between male and female), *p ≤ 0.05, **p ≤ 0.01, ***p ≤ 0.001, ****p ≤ 0.0001.

The metabolic activity of human chondrocytes was measured by water‐soluble tetrazolium salt (WST‐1) assay, whereby a high optical density (OD) correlates with a high metabolic activity of the cells. As shown in Figure 3a, following stimulation with 0.8–1.2 V/m (mean ± SD: 0.14 ± 0.04, *p* = 0.0915) and 15–20 V/m (mean ± SD: 0.15 ± 0.03, *p* = 0.0830), the metabolic activity revealed a decreasing trend compared to the control group (mean ± SD: 0.20 ± 0.08). Comparisons within and between chondrocytes from male and female donors revealed no differences in cell metabolic activity (Figure 3b).

### Collagen protein synthesis

**Figure 4:**
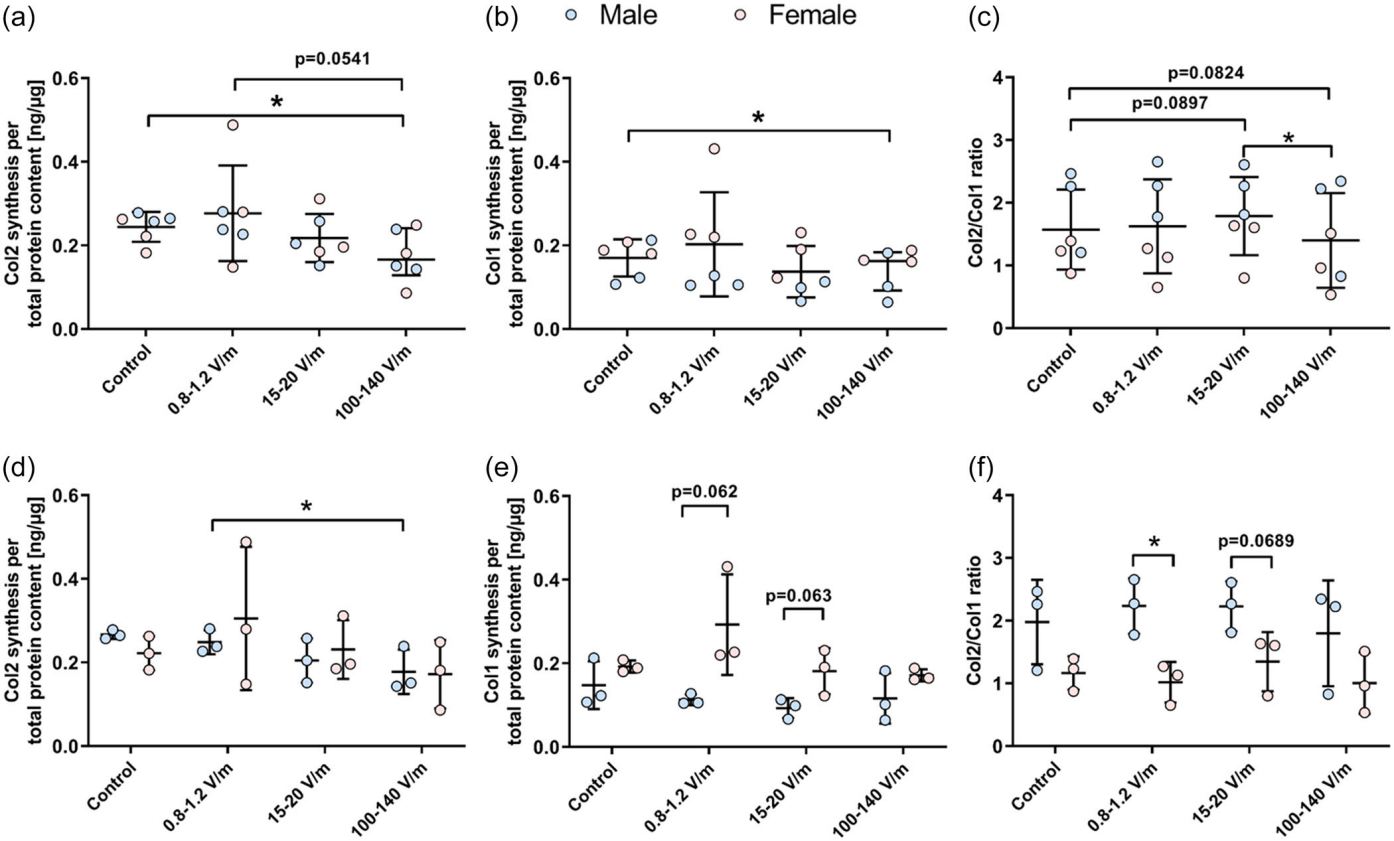
Human chondrocytes were exposed to alternating electric fields of 0.8–1.2, 15–20 and 100–140 for 3 days. Subsequently, the synthesis of collagen (Col)2 and Col1 was assessed in the supernatant and normalized to total protein content. (a) Comparison of Col2 synthesis rates between different groups. (b) Comparison of Col1 synthesis between different groups; (c) Col2/Col1 ratio was calculated and compared between different groups. (d) Comparison of Col2 synthesis both within and between chondrocytes from male and female donors. (e) Comparison of Col1 synthesis both within and between chondrocytes from male and female donors. (f) The Col2/Col1 ratio was calculated separately for males and females and then compared both within and between chondrocytes from male and female donors. All data were presented as individual values with mean and standard deviations. Statistical analysis was conducted using a paired *t*‐test. An unpaired *t*‐test was performed to compare males and females. *p ≤ 0.05.

The quantification of Col1 and Col2 in the supernatant was conducted to determine the synthesis rate of ECM proteins following electrical stimulation (Figure 4).

In comparison to the control group, the electrically stimulated groups exhibited the following changes. 0.8–1.2 V/m: No significant changes were observed in the synthesis of Col1 and Col2, as well as the Col2/Col1 ratio (Figure 4a–c). Following stimulation with 15–20 V/m, no alterations were noted in Col2 and Col1 synthesis (Figure 4a,b). However, an ascending trend (*p* = 0.0897) in the Col2/Col1 synthesis ratio in this group (mean ± SD: 1.79 ± 0.62) was observed compared to the control group (mean ± SD: 1.57 ± 0.64) (Figure 4c). Stimulation with 100–140 V/m resulted in a significant reduction in Col2 (mean ± SD: 0.18 ± 0.06 ng/µg, *p* = 0.0180; Figure 4a) and Col1 (mean ± SD: 0.14 ± 0.05 ng/µg, *p* = 0.0493; Figure 4b) compared to the control group. The Col2/Col1 synthesis ratio (mean ± SD: 1.40 ± 0.76) showed a decreasing trend (*p* = 0.0824) compared to the control group (mean ± SD: 1.57 ± 0.64; Figure 4c). Additionally, a comparison between electrically stimulated groups revealed that the Col2/Col1 synthesis ratio following stimulation was significantly higher with 15–20 V/m (mean ± SD: 1.79 ± 0.62, *p* = 0.0489) compared to 100–140 V/m (mean ± SD: 1.40 ± 0.76; Figure 4c).

Furthermore, comparisons were made both within and between chondrocytes from male and female donors (Figure 4d–f). The comparison revealed a higher Col2 synthesis following stimulation with 0.8–1.2 V/m (mean ± SD: 0.25 ± 0.03, *p* = 0.0444) compared to the 100–140 V/m group (mean ± SD: 0.18 ± 0.05) in chondrocytes derived from male donors (Figure 4d). Comparisons between both sexes showed that following stimulation with 0.8–1.2 V/m (*p* = 0.0615) and 15–20 V/m (*p* = 0.0630), chondrocytes from female donors exhibited a higher trend of Col1 synthesis (Figure 4e). Conversely, there were no significant differences in Col2 syntheses between chondrocytes from male and female donors. Consequently, after stimulation with 0.8–1.2 V/m, chondrocytes from female donors exhibited a significantly lower Col2/Col1 ratio (mean ± SD: 1.02 ± 0.32, *p* = 0.0185) compared to chondrocytes from male donors (mean ± SD: 2.23 ± 0.44). Following stimulation with 15–20 V/m, a decreasing trend (*p* = 0.0689) of the Col2/Col1 ratio of chondrocytes from female donors compared to those from male donors was observed (Figure 4f).

## DISCUSSION

Despite promising results, determining the optimal parameters for the electrical stimulation of chondrocytes remains a challenge. In the literature, the use of sinusoidal alternating fields with a frequency of 60 kHz was proposed for the stimulation of chondrocytes [4, 5, 50, 53]. Furthermore, Vaca‐González et al. [51] investigated the influence of various parameters, including stimulation time, output frequencies, voltages and electric field strengths. In our current study, a frequency of 20 Hz was also used, which was rarely applied in human chondrocyte cultures in vitro but has shown promising results in promoting bone regeneration [12, 41]. At the same time, in vitro studies into electrical stimulation encompass a wide range of electric field strengths, from as low as 5.2 × 10^−6^ V/m [28] to as high as 35 V/m [19]. The most common electric field strength of 2 V/m, proposed by Brighton et al. [6, 7, 53], was reported to increase the production of Col2 and proteoglycans, as well as the upregulation of their mRNA expression. In contrast, Vaca‐González et al. [50] suggested that the electric field strength should range between 0.4 and 0.8 V/m. Moreover, electric field strengths of 20–35 V/m have been used on human chondrocytes and mesenchymal stem cells, resulting in increased synthesis of Col2 under hypoxic conditions [19]. Higher field strengths, such as 80–90 V/m, have not yet been reported for in vitro stimulation of chondrocytes but have been used for electrical stimulation of osteoblasts and bacteria [12].

In our current study, the stimulation setup was based on the construction of a previous study from Hiemer et al. [19]. Stimulation parameters such as sinusoidal signal form and stimulation time are selected based on earlier investigations within our working group [12, 19, 42]. Opting for alternating current (AC) stimulation over direct current (DC) stimulation is aimed at minimizing the potential adverse electrochemical reactions associated with DC stimulation [19]. Compared to previous works, we used electric fields of 0.8–1.2, 15–20 and 100–140 V/m for stimulation of human chondrocytes in the current experiments to evaluate their influence on cell morphology and differentiation.

It is widely acknowledged that dedifferentiated chondrocytes transition from secreting Col2, Col9, Col11, and aggrecan to Col1 and Col3, lose their rounded structure, adopting a fibroblastic morphology, precisely an elongated form [14, 31, 33, 35]. We used the form factor as the shape descriptor of chondrocytes. Studies have shown that during the dedifferentiation process of chondrocytes, the form factor of chondrocytes decreases [37, 38], whereas inhibiting the dedifferentiation allows chondrocytes to regain their spherical shape [39]. In our experiments, an increase in the proportion of rounded chondrocytes under electrical stimulation of 15–20 V/m was observed. This is consistent with the increasing trend of the Col2/Col1 ratio, suggesting that an electric field of 15–20 V/m might have a promoting effect on the redifferentiation of chondrocytes. Additionally, research has shown that helping chondrocytes maintain their round morphology allows them to exhibit greater replication capacity, resulting in higher quality and quantity of new cartilage formation in vitro [46]. This suggests that maintaining the rounded morphology of chondrocytes may also have some positive implications for hyaline cartilage production during ACI.

The exploration of electrical stimulation on chondrocyte ECM synthesis has also yielded observations in Col1 and Col2 synthesis rates. Among the three experimental groups stimulated by 0.8–1.2, 15–20 and 100–140 V/m, a decrease in the synthesis of Col1 and Col2 was only observed after 100–140 V/m electrical stimulation, with no statistically significant changes observed in the other two groups. However, compared to the isolated analysis of Col1 and Col2, analysing the ratio of Col2/Col1 provides a more in‐depth understanding of the progression of chondrocytes toward differentiation processes [2, 36]. Previous studies have shown that the Col2/Col1 ratio can significantly decrease, from 15‐fold to over 1800‐fold, within 10 days in monolayer culture, indicating cellular dedifferentiation [2, 36]. In our present study, following the stimulation of 15–20 V/m, an ascending trend in the Col2/Col1 ratio was observed. Conversely, under the stimulation of 100–140 V/m, the Col2/Col1 ratio exhibited a descending trend. These results are consistent with the cell morphology observation, implying that electric fields of 15–20 V/m might promote the redifferentiation of chondrocytes, while 100–140 V/m might inhibit the redifferentiation of chondrocytes. In a previous study by Hiemer et al. [19], electrical stimulation was applied to human chondrocytes, bone marrow‐derived mesenchymal stem cells (BM‐MSC), and co‐cultures of chondrocytes with BM‐MSC under both hypoxic and normoxic conditions. The electric field strengths used were 20–35 V/m, which was close to the 15–20 V/m in our current experiments. Hiemer et al. [19] indicated that under normoxic conditions, no significant changes were observed in the synthesis of Col1 and Col2 following electrical stimulation. However, under hypoxic conditions, an increase in the Col2/Col1 ratio of stimulated chondrocytes was noted. Although the rise in the Col2/Col1 ratio in their experiment was primarily attributed to an increase in Col2 synthesis, a phenomenon not observed in our study, the outcomes from both studies suggest that electric field strengths within a similar range may have an effect to inhibit the dedifferentiation or promote redifferentiation of human chondrocytes.

Human chondrocytes following electrical stimulation of 0.8–1.2 and 15–20 V/m showed a slightly diminishing trend in cell metabolic activity. However, in previous experiments that also used direct coupling to deliver 20–35 V/m electric fields on human chondrocytes and mesenchymal stem cells, no changes in cell metabolic activity were observed [19]. Theoretically, changing the direct coupling to capacitive coupling can prevent the adverse effects of electrochemical reactions by avoiding direct contact with electrodes and electrolytes [28, 49]. However, a decrease in metabolic activity of human chondrocytes was also observed by Krueger et al. [28] when using capacitive coupling, with increased synthesis rates of Col2 and glycosaminoglycans noted simultaneously. Therefore, the decrease in metabolic activity of chondrocytes may be attributed to other reasons other than electrochemical changes. It has been reported that during the initial phase of dedifferentiation, chondrocytes exhibit a glycolytic phenotype with heightened expression of metabolic and antioxidant‐related genes, leading to increased cellular activity [11]. We suggest that the observed reduction in cellular activity might be indicative of reduced chondrocyte dedifferentiation. Upon comprehensive observation of the OD values and Col2/Col1 ratio of electrical stimulation groups, a particular inverse relationship was observed. Specifically, the only electrical stimulation group that did not show reduced cellular metabolic activity, which is the 100–140 V/m electrical stimulation group, exhibited a decrease in the Col2/Col1 ratio. Meanwhile, a reduction in the Col2/Col1 ratio implies an enhancement in the trend of dedifferentiation [2, 36].

Another intriguing aspect of this experiment is the sex disparity observed in chondrocytes. Comparison between chondrocytes from males and females revealed significantly higher Col1 synthesis and lower Col2/Col1 ratios in cells from female donors, especially after electrical stimulation with the lower electric fields of 0.8–1.2 and 15–20 V/m. At the same time, these cells display a higher proportion of elongated chondrocytes following in vitro culture. Instead, most of the cells from male donors exhibited a more roundish morphology. Although exposure to the aforementioned electric fields induces a rounder phenotype in cells from females (form factor between 0.8 and 1.0: 42.0% [control]; 47.7% [0.8–1.2 V/m]; 52.5% [15–20 V/m]; 41.1% [100–140 V/m]), chondrocytes from male donors show a substantially higher proportion of rounded and, thus re‐differentiated cells (form factor between 0.8 and 1.0: 56.6% [control]; 60.4% [0.8–1.2 V/m]; 63.7% [15–20 V/m); 53.7% [100–140 V/m]).

Other studies revealed further that chondrocytes derived from female donors showed significantly lower gene expression and protein synthesis of SRY‐box transcription factor 9 (SOX9) compared to chondrocytes from male donors [22]. Similar sex disparities were also observed in bovine chondrocytes [18]. Meanwhile, the literature indicates that SOX9 plays a role in inhibiting the dedifferentiation of chondrocytes [17], and SOX9 transduction can significantly reduce the expression of Col1 [48]. That might explain the higher levels of Col1 synthesis observed in chondrocytes from female donors in our study, suggesting that these cells have a higher tendency toward dedifferentiation compared to males.

Taking into account the age distribution in both sexes, our study showed that the female cell donors were older compared to the males. Despite these age differences, studies have shown that the age‐related differences in the synthesis of matrix macromolecules such as proteoglycans and collagen are primarily due to changes in the number of chondrocytes at different ages. However, an equal number of chondrocytes synthesized similar amounts of matrix macromolecules regardless of whether they originated from younger or older cartilage tissue [3]. In our experimental study, equal numbers of chondrocytes were used in each male and female group, so the differences in protein secretion are not attributed to differences in cell numbers. Moreover, no significant Pearson correlations were found between age and Col1 (control: *p* > 0.9999; 0.8–1.2 V/m: *p* = 0.1028; 15–20 V/m: *p* = 0.1750; 100–140 V/m: *p* = 0.8028), Col2 (control: *p* = 0.2775; 0.8–1.2 V/m: *p* = 0.2014; 15–20 V/m: *p* = 0.9538; 100–140 V/m: *p* = 0.8172) and the ratio of Col2 to Col1 (control: *p* = 0.3904; 0.8–1.2 V/m: *p* = 0.2457; 15–20 V/m: *p* = 0.1820; 100–140 V/m: *p* = 0.5833) in all electrical stimulation and control groups. Therefore, our observed data are more likely due to sex differences. Genetic studies revealed that female cartilage shows predominantly molecular changes related to ECM degradation in response to OA. In contrast, male cartilage primarily shows no significant ECM degradation [30]. This is consistent with the results we found. A similar phenomenon was observed in osteoblasts, where a significant reduction in the ability to synthesize collagen was not evident in older men, whereas the synthesis of pro‐collagen may be reduced in older women [1].

Besides chondrocytes, similar sex differences were also reported in human mesenchymal stem cells [43]. In addition to higher expression levels of specific growth factors involved in bone regeneration and defined osteogenic markers in male MSCs, Salamanna et al. [43] also observed higher SOX9 expression, suggesting not only greater osteogenic but also more remarkable chondrogenic differentiation ability in male MSCs. That is consistent with the higher chondrogenic differentiation capacity observed in chondrocytes from male donors. The higher tendency to de‐differentiate implies a greater likelihood of producing fibrocartilage with inferior mechanical properties during in vitro expansion in the case of ACI [9]. Therefore, this observation led to the hypothesis of whether the postoperative outcomes of ACI might be less favourable in females compared to males. Consulting literature data on the differences between the sexes in clinical outcomes after ACI, Filardo et al. [15] evaluated 250 knees treated with matrix‐assisted ACI (MACI) at one, two and a minimum of five years of follow‐up. This evaluation demonstrated superior subjective International Knee Documentation Committee (IKDC) scores in male patients compared to female patients at all follow‐up intervals. Similarly, Kreuz et al. [26] followed up 52 patients who underwent ACI at 6, 12 and 48 months postoperatively, observing significantly better knee injury and osteoarthritis outcome (KOOS) scores and higher strength values in male patients during the follow‐up period. These follow‐up results, which show consistency with our hypothesis, imply that the difference in dedifferentiation tendency between chondrocytes from male and female during in vitro expansion might be one of the reasons for the differing postoperative outcomes of ACI between different sexes.

However, our present study has some limitations. Due to the limited number of donors in our present study, only three groups from each sex could be assessed. Hence, future studies should include more samples of chondrocytes from male and female donors to verify the observed differences in sex. In addition, this study only assessed Col2 and Col1 as markers of chondrocyte redifferentiation and dedifferentiation. In future experiments, more redifferentiation markers such as aggrecan and SOX9, as well as the dedifferentiation markers such as matrix metalloproteinase‐13, will be evaluated to more comprehensively analyse the regulatory effects of the electric field on the differentiation of human chondrocytes. In addition to inherent differences in redifferentiation capacity based on sex, it was observed that electrical stimulation exhibited a more significant redifferentiation‐promoting effect on chondrocytes derived from male donors. In future experiments, the stimulation period should be extended to verify whether there is still a difference in the response of chondrocytes from male and female donors to electrical stimulation. In addition, the influence of alternating electric fields on sex‐specific redifferentiation in a physiologically approximated three‐dimensional cultivation could be adapted in further work. This should also focus on the comparison of a natural three‐dimensional matrix of chondrocyte spheroids or cultivation in synthetic hydrogel structures. Both would have the advantage that the deposition of important matrix proteins in relation to either the electric field strength or sex can be specifically detected.

In conclusion, electric field strengths in the range of 15–20 V/m show a promotive effect on the redifferentiation of human chondrocytes. Although electrical stimulation exhibited a more significant redifferentiation‐promoting effect on chondrocytes derived from male donors, it remains unclear whether extending the duration of electrical stimulation can eliminate this distinction. Future research should also focus on an in‐depth exploration of its mechanism, especially its effect on the signalling pathways governing ECM synthesis. Concurrently, we observed that chondrocytes derived from the articular cartilage of female donors seem to exhibit a stronger tendency to de‐differentiate during in vitro cultivation. Future studies should include more chondrocytes from male and female donors to verify the observed differences in sex further. Given the limited research on the age‐related differences in osteoarthritic cartilage from elderly patients/donors, we further plan to use chondrocytes from male and female donors of similar age within an in‐vitro study to further verify our current results, while also exploring the impact of age by using chondrocytes from donors from the same sexes but different ages.

## AUTHOR CONTRIBUTIONS

**Zezhong Song**: Conceptualization; investigation; validation; writing—original draft preparation. **Vivica Freiin Grote**: Formal analysis; methodology; data curation; writing—original draft preparation; supervision. **Franziska Sahm:** Formal analysis; supervision; writing—review and editing. **Julius Zimmermann:** Methodology; validation; writing—review and editing. **Christoph Lutter**: Resources; supervision; writing—review and editing. **Anika Jonitz‐Heincke**: Project administration; resources; data curation; supervision; formal analysis; validation; writing—original draft preparation. **Rainer Bader**: Project administration; resources; supervision; writing—review and editing.

## CONFLICT OF INTEREST STATEMENT

The authors declare no conflicts of interest.

## ETHICS STATEMENT

The study was conducted with the approval of the Local Ethical Committee of the University of Rostock (registration no. A2009‐17). Informed consent was obtained from all subjects involved in the study.

## Data Availability

The data presented in this study are available on request from the corresponding authors.
